# Supplementary material for: Are carbon emissions trading and green financial instruments synergistic? -Comprehensive quantitative research based on content analysis
Source: PLoS One. 2024 Mar 7;19(3):e0298601. doi: 10.1371/journal.pone.0298601 (PMC10919601; doi:10.1371/journal.pone.0298601)
Supplement: S1 Data — (ZIP) [file pone.0298601.s001.zip › Data/地级市Z_F工作报告.zip!/─┌├╔╣┼/░n═╖╩╨/└┤╫╘╣1⁄2╓┌║┼í╛┬φ┐╦╩2╛▌═°í┐_2013.docx]

各位代表：

　　现在，我代表市人民政府，向大会报告工作，请予审议，并请市政协各位委员和其他列席人员提出意见。

　　过去五年工作回顾

　　五年来，在自治区党委、政府和市委的领导下，在市人大、市政协的监督、支持下，市政府团结依靠全市各族人民，大力实施“科学发展、全面转型、富民强市”战略，完成了市第十三届人民代表大会历次会议确定的各项任务，经济社会实现新发展，全面小康社会建设取得新成绩。

　　———综合实力大幅提升。紧紧抓住国家实施西部大开发战略的历史机遇，认真落实《国务院关于进一步促进内蒙古经济社会又好又快发展的若干意见》，按照自治区促进优势地区率先发展的要求，坚定信心，攻坚克难，经济实现了平稳较快发展，经济总量跨越了两千亿元和三千亿元两个台阶。预计2012年全市生产总值完成3440亿元左右，是2007年的2.1倍；规模以上工业增加值达到1300亿元以上，是2007年的2.5倍；社会消费品零售总额完成960亿元以上，年均增长17.6%；固定资产投资五年累计完成9091亿元，年均增加349亿元。地方财政总收入完成326.8亿元，是2007年的2.3倍。这五年是发展持续加快、经济总量大幅跃升的五年。

　　———产业结构不断调整。坚持一产做精、二产做强、三产做优，集约集群发展不断加快。新型工业化加速推进。围绕优化工业结构，有效提升产业层次。钢铁、铝业、装备制造等五大支柱产业不断做大做强，预计完成增加值910亿元左右，是2007年的2.5倍。装备制造业增加值占规模以上工业的比重达到22%，比2007年提高近十个百分点。煤化工、风力发电等战略性新兴产业快速发展，产值已占规模以上工业的11.7%。世界首套煤制烯烃示范项目顺利投产、运营良好，风力发电装机容量达到115.7万千瓦。围绕增强工业经济实力，大力实施“双百亿工程”。营业收入超百亿元企业达到六户，包钢集团成为自治区唯一超500亿元的工业企业；营业收入超百亿的园区达到七个，稀土高新区在自治区率先进入千亿元级园区行列，成为全国首批五个创新型特色园区之一。围绕提升工业竞争力，着力增强自主创新能力。拥有国家、自治区级工程中心和企业技术中心四十四个，高新技术企业达到七十三家。高新技术产业预计实现产值750亿元，是2007年的2.2倍。大口径厚壁无缝钢管垂直挤压设备研制成功，打破了国外技术垄断；高速百米重轨、大功率自动变速箱、核磁共振成像系统达到国内先进水平；北奔重汽成为我国西部重要的重型汽车生产基地，北方股份矿用车通过引进消化再创新，市场占有率国内领先。服务业活力显著增强。一批国内外知名商业企业入驻包头，一批本土企业发展壮大。“三基地、六园区、九中心”物流业发展规划出台实施，北方国际工业原料城、润恒物流园等物流重点项目加快建设，现代物流业迅速崛起。区域性商贸中心建设有力推进。房地产业平稳健康发展，直接或间接纳税220多亿元。金融业发展提速，六家股份制银行落户包头，包商银行不断壮大，金融对地方经济发展的支持力度进一步加大。会展业开始起步，国际会展中心建成运营，稀土论坛的影响力继续扩大，包头被命名为“稀土之都”。旅游业快速发展，接待人数年均增长13.1%，旅游收入年均增长23.5%。中介、法律、咨询、会计等现代服务业快速推进。现代农牧业长足发展。大力实施“南菜北薯、乳肉并举”战略，蔬菜保护地增加到13.5万亩，年均增长35%；马铃薯等滴灌设施面积达到40万亩，年均增长2.3倍；奶牛、肉羊的养殖规模和水平不断提高。积极推进农牧业产业化，市级以上龙头企业由42家增加到101家，农畜产品加工转化率由40%提高到50%。农牧业科技装备水平和耕种收综合机械化水平稳步提高。这五年是经济素质整体提升、产业竞争力不断增强的五年。

　　———城市功能日臻完善。强化规划编制，完成了六个独立工矿区总规及城市色彩规划编制，实现了对全市建设用地的控规全覆盖。邀请国内外知名设计团队设计，聘请专家把关，完成了钢铁大街和阿尔丁南大街等十一个重点区域的城市设计。新都市中心区、昆河沿岸和昆区南部区、滨河新区、石拐新区等重点区域建设有序推进，新城市规划展览馆建成使用。加强城市建设，实施了740余项城建项目，累计投入近千亿元。完成了青山路等100多条道路新建改扩建，人均拥有道路面积13.5平方米，比2007年提高了2.7平方米。新建、改造东华、希铝电厂等供热设施及城市供热干网，供热普及率达到91%，提高了15个百分点。推进天然气门站、输配气管网建设，燃气普及率达到95.5%，提高了10.8个百分点。垃圾无害化处理率达到93%，提高了两个百分点。实施了给排水工程，城市日供水能力达到120万立方米，增加29万立方米；排水管网长度达到2062公里，增加639公里。加快北郊、东河东等污水处理厂新建、改扩建，污水厂集中处理率达到85%，再生水利用率达到45%，分别提高了3.4个和31.2个百分点。建成稀土公园、锦绣公园等主题公园，改造建设路、阿尔丁大街等主要干道景观，建成区绿化覆盖率达到42%，提高了5.4个百分点。成立综合执法部门，完善管理长效机制，城市管理水平进一步提高。加快综合交通体系建设，实施了110国道包头段、210国道城区段等公路工程，京藏高速包头段北幅通车。累计完成投资130亿元，公路总里程达4207公里。加快输变电主网建设，初步实现电网升级换代。包头至西安铁路和集包第二双线开通，包满铁路竣工，机场迁建项目前期工作有序展开。这五年是基础设施建设投入最大、承载功能不断提升的五年。

　　———城乡统筹扎实开展。财政用于惠农的支出达到98亿元，年均增长27.7%。全市农村牧区危旧房改造1.1万户，建成农村牧区公路3287公里，解决了24.64万人的安全饮水和6.81万贫困人口的生产生活问题。新农村新牧区建设试点村基本实现了通油路、通有线电视、有卫生室等目标。组织开展集中社会力量重点帮扶固阳县工作，帮扶资金物资达到2.3亿元，实施重点项目662个，有力推动了固阳县经济社会发展。有效破解旗县区发展难题，加大了财政转移支付力度，适时调整了行政区划，实施了山北地区给水工程和一级公路通达工程。引水入白和包头至固阳输供水工程建成通水，包头北出口至固阳明登公路运营，包头至石拐、固阳至百灵庙等一批通旗县区一级公路建设加快。制定城乡一体化规划，实现了村镇规划全覆盖。

出台了户籍管理、社会保障、土地利用等指导性意见，为城乡一体化发展奠定了坚实基础。这五年是“三农”工作显著加强、一些影响农牧民生产生活的突出问题得到有效缓解的五年。

　　———环境保护和生态建设力度加大。实施“蓝天、绿水、青山、大草原”工程，强化“三片两线”环境综合整治。单位生产总值能耗累计下降25.9%，节能减排工作完成自治区目标。包头黄河湿地公园作为国家湿地公园建设试点，列入国家“十二五”发展规划。黄河包头段三断面水质平均达标率由75%提高到100%。大青山生态供水工程全面竣工，小白河蓄滞洪区建设基本完成，实现分洪蓄水，初步形成了“南北贯通、东西互济”的供水格局。推进国家重点林业工程，完成造林任务247.7万亩。实施大青山南坡绿化一期、二期工程及城郊结合部和村屯绿化工程，累计造林16万亩。全市森林覆盖率由12.4%提高到16.3%。设立大青山国家自然保护区，实施了一批生态修复工程，取缔整合了一批采碎石企业，区域环境得到有效保护。率先在牧区草场实施阶段性禁牧，成为自治区唯一实现禁牧和补贴全覆盖的盟市。山北地区草场植被平均盖度达到31%，较禁牧前提高9.6个百分点。这五年是生态文明建设不断加强、城乡环境逐步改善的五年。

　　——改革开放不断深化。加大农村牧区土地流转力度，进一步完善水权转让制度，基本完成集体林权改革确权发证工作。国有企业改革不断推进，七十六户企业改革重组，八万多职工妥善安置。国有企业厂办大集体改革全面启动，国企职教幼教退休教师待遇问题得到解决。非公有制经济不断壮大，占全市经济的份额超过50%，贡献了70%以上的税收，解决了90%以上的新增就业。深化行政审批制度改革，取消、调整审批事项104项。不断完善财政体制，用足用好积极的财政政策，累计争取上级资金430多亿元。深入推进事业单位人事制度改革，不断深化职称制度改革，事业单位岗位设置管理和绩效工资制度率先入轨。文化体制改革深入实施，文艺院团活力有所增强。扎实推进医药卫生体制改革，五项重点任务实现了阶段性目标。外向型经济发展加快，满都拉口岸基础设施逐步完善，累计进出口货物突破50万吨，“无水港”完成货运量突破200万吨。五年引进国内（区外）资金1702亿元，利用外资51.7亿美元，进出口总额实现103.4亿美元。这五年是各项改革深入推进、发展动力和活力不断增强的五年。

　　——人民生活显著改善。财政用于民生的支出达到800多亿元，占地方财政支出的比重提高了十一个百分点。预计2012年城乡居民收入分别达到33480元左右和11370元左右，是2007年的1.9倍和1.8倍。累计实施好事实事项目181项，切实解决了一批人民群众关注的热点难点问题。实施积极的就业政策，城镇新增就业24.2万人，其中大学生五万多人，城镇登记失业率控制在4%以内。社会保障体系不断完善，基本养老和医疗保险实现制度全覆盖。保障标准不断提高，城镇居民医疗保险财政补助标准由70元提高到240元，企业退休人员月人均养老金由1095元提高到1889元。城市低保月保障标准由230元提高到505元，农村牧区低保年保障标准由540元提高到4480元。“三无人员”、“三民”、“孤儿”生活补贴标准，城乡困难群体临时物价补贴标准，城镇低保户取暖补贴标准进一步提高。在全区率先发放高龄津贴，累计为80岁以上老人发放5060万元。我市初步建立了以居家养老为基础、社区养老服务为依托、机构养老为支撑的养老服务体系。居住条件持续改善，累计投入5.2亿元，治理旧小区185个、改造面积1400多万平方米。石拐棚户区搬迁完成，包钢尾矿坝周边五村搬迁加快推进。开工建设保障性住房24.2万套，完工13.2万套。这五年是人民群众得到实惠更多、共享发展成果更充分的五年。

　　———社会建设全面推进。教育事业不断发展。累计投入150多亿元，加快校安工程和校园标准化建设，新建、改造校舍280多万平方米。积极推进义务教育均衡发展，素质教育成效显著。教师队伍建设逐步加强，教育教学水平持续提高，高考成绩名列自治区前茅。整合职业教育资源，职教园区一期工程全面完工，六所中高职学校即将入住。成功举办了世界中学生排球锦标赛、第十一届全国中学生运动会。文化事业不断繁荣。新图书馆和少年宫投入使用，金融文化中心加快实施。四级公共文化服务网络基本建立。打造了“鹿城文化艺术节”、“内蒙古二人台艺术节”等一批特色节庆品牌，创作了话剧《牛玉儒》、舞剧《额吉》、漫瀚剧《草原阿妈》等文艺精品，丰富了群众文化生活。电影《吴运铎》荣获中宣部“五个一工程奖”。“包头剪纸”、“梅日更召信俗”入选第三批国家级非物质文化遗产名录。广播电视工作不断加强，广播、电视混合覆盖率提高到98%以上。城乡医疗卫生服务体系进一步完善。新型农村牧区合作医疗率先在全国实现市级统筹，人均补助标准由40元提高到258元，参合率提高到98.9%。低生育水平持续稳定，人口计生公共服务体系不断完善。优抚标准不断提高，退伍安置工作持续加强，实现了全国双拥模范城“七连冠”和自治区双拥模范城“八连冠”。大力加强人才工作，智力支撑和人才保障作用不断强化。着力推进精神文明建设，第三次荣获全国文明城市称号。首次进入国家卫生城市行列，再次荣获全国民族团结进步模范集体荣誉称号，进入国家科技进步示范市行列，成为自治区首个全国节水型社会建设示范市。全面推进和谐矿区建设，依法规范了用地和矿产勘查开发秩序。加强和谐社区建设，创新社会管理，“精街道强社区”试点工作取得阶段性成果。累计投入1.2亿元，建成300平方米以上的社区活动中心185个，占社区总数的74%。着力加强信访工作，不断解决群众反映强烈的突出问题。深入开展社会治安综合治理，严厉打击各类刑事犯罪。应急体系建设不断完善，妥善处置了各种突发事件。坚持标本兼治，安全生产水平稳步提高。严格市场监管，加大专项整治，确保产品质量和食品药品安全，维护了人民群众的合法权益。妇女儿童、老年人、残疾人事业进一步加强，统计、人防、气象、地震、外事、史志、档案、仲裁、红十字会等工作取得新成绩。这五年是公共服务不断完善、社会事业繁荣发展的五年。

　　在推进经济社会发展的过程中，我们始终重视政府自身建设。认真执行市人大及其常委会的决议、决定，自觉接受市人大及其常委会的法律监督、工作监督和市政协的民主监督。五年间，市政府及有关部门办理人大代表建议869件、政协提案1904件。深入推进政府职能转变，加快电子政务建设，大力实施政务公开和事务公开，增强了政府工作的透明度。改进目标考评办法，强化政务督查，有效提升了政府执行力。加强调查研究，实行重大事项听证制度，提高了政府决策的科学化、民主化水平。全面推进依法治市，依法行政意识进一步增强，第三次荣获全国法制宣传教育先进市称号。加强行政监察和审计工作，廉政建设和反腐败工作深入开展。强化对公务员的素质提升和管理监督，政府公共服务能力不断提高。

　　各位代表！2012年，宏观经济形势复杂多变、不利因素相互交织，成为2008年以来最为困难的时期。面对压力和困难，我们坚持把稳增长作为首要任务，把促转型作为工作主线，把惠民生作为根本目的，充分用好政策机遇，有效破解发展难题，切实增强应对能力，牢牢把握发展的主动权，基本完成了年初确定的主要目标任务。

　　在外部环境不利、经济下行压力加大的情况下，经济实现平稳健康发展。针对国际金融危机的持续影响，我们多措并举，千方百计保持经济平稳运行。出台了《稳增长、促发展确保经济平稳较快发展若干意见》，在项目建设、产业发展、实体经济等十二个方面制定了扶持措施。设立专项基金支持中小企业贷款贴息和电价补贴。采取房产交易展示、推出优惠政策等措施，推动房地产业健康发展。加大对生产运行的指导，不断做好服务工作。深入旗县区，了解实际情况，及时掌握第一手资料，研判经济形势，把握发展走势。深入企业，及时解决企业发展过程中的矛盾和问题，支持企业技术研发、开发新产品和开拓市场，有效推动实体经济发展。深入有关部门，督促检查项目审批等方面的问题，规范和简化手续，提高办事效率。加大招商引资力度，一批大项目成功签约落地。投资近500亿元的包钢综合技改工程加快实施，投资200亿元的中国兵器高端装备制造园建设加快，五万台北奔重卡总装、十万台重卡车架等项目的土建工程基本完工，十万台重卡发动机合作项目基本确定。泛海煤化工、包铝自备电厂和扩能技改、华电2×60万千瓦电厂、包头常铝铝深加工、包钢稀土高性能永磁材料二期、亚新隆顺钢铁技改、达茂旗巴音百万千瓦级风电基地等项目进展顺利。全市经济运行平稳，预计地区生产总值增长12.5%以上，高于全国和全区平均水平。

　　在经济增速放缓、节能减排约束趋紧的情况下，转变发展方式取得积极成效。从优化环境入手，以“三片两线”综合整治为重点，继续关闭煤焦化企业、拆除小高炉，关停、改造部分旧有设备，加快推进锅炉烟气脱硫除尘。进一步完善城市设计，重点区域部分工程已开始实施。积极实施“十二五”文化改革发展规划纲要，完成了赛汗塔拉草原蒙元文化、南海湖西口文化、九原秦汉文化等重点文化产业园区的规划设计。积极推进五当召景区改造，黄河龙城项目、国家广告产业园项目成功落地。加快引智步伐，包钢集团与东北大学、北重集团与清华大学的产学研合作全面深化。北京大学联合海内外科研机构建设的研究院和科技园开工奠基。稀土高新区与北京中关村共建的高新技术产业园区加快推进。包头市云计算应用中心在全区率先投入商业运营。全国第一家稀土产品交易平台“包头稀土产品交易所”正式揭牌。

　　在财政增收难度加大、刚性支出增长的情况下，民生得到有效改善。加快公立幼儿园建设，新建、改扩建三十个，“入园难”问题有所缓解。改造了集贸市场十个，累计达到十八个，方便了群众生活。建立地方储备制度，增强了市场应急调控能力。既有建筑节能改造完成200万平方米，累计达到800万平方米，惠及家庭近十万户。健康水受益人口新增10.12万人，累计达到143.36万人。坚持公交优先，购置车辆和建设公交站场，优化公交线路。新建改造黄河大街、兵工大道等十七条道路，八个路口渠化全部完成，方便了群众出行。

　　在黄河特大洪峰突如其来、防线长任务重的情况下，抗洪工作取得全面胜利。去年七月下旬，黄河发生建国以来罕见的特大洪水。面对严峻汛情，市委、市政府果断决策、周密部署，广大干部群众和人民子弟兵紧急动员、迅速集结，投入到这场保护人民生命财产安全、保卫我市经济建设成果的重大战役。经过全体军民近六十天的奋战，赢得了黄河抗洪抢险的胜利。“以人为本、为民负责，万众一心、众志成城，不畏艰难、顽强拼搏，坚韧不拔、勇于胜利”的伟大抗洪精神，成为激励全市各族人民建设包头、发展包头的不竭动力。

　　各位代表：五年来，我们深入贯彻落实科学发展观，开拓创新，真抓实干，开创了经济社会发展的新局面。我们深深感到：这是认真贯彻国家、自治区一系列方针政策，全面落实市委的工作部署，解放思想、与时俱进的结果；这是坚持结构调整，大力推进环境保护和生态建设，加快转变经济发展方式的结果；这是坚持发挥内力和借助外力相结合，全方位扩大对外开放，不断解放和发展社会生产力的结果；这是更加重视民生，从解决人民群众的现实利益问题入手，不断加大投入并取得积极成效的结果。在此，我代表市人民政府，向全市各族干部群众，向人大代表、政协委员，向离退休老同志，向各民主党派、工商联、人民团体、社会各界，向中央和自治区驻包单位，向驻包解放军、武警官兵和公安干警，向所有关心支持包头建设的同志们、朋友们，表示崇高的敬意和衷心的感谢！

　　回顾五年来的工作，我们清醒地认识到，包头经济社会发展还存在不少困难和问题，面临诸多压力和挑战。主要表现在：产业层次不高，转型升级的任务还比较重；文化产业发展不快，还不能有效满足人民群众日益增长的文化需求；科技投入不足，科技成果转化能力还不够强；资源环境约束日益凸显，节能减排压力还比较大；城市承载功能有待完善，公共服务能力还需进一步提升；城乡统筹步伐还比较慢，居民收入分配差距依然较大；有些部门和工作人员作风漂浮、行政效能不高，等等。这些问题必须引起我们的高度重视，有针对性地采取措施，认真加以解决。

　　今后五年工作的总体要求和2013年的主要任务

　　今后五年，是推动包头全面转型的重要时期，是提前全面建成小康社会的关键阶段。党的十八大为我们指明了前进方向，提供了强大的思想理论武器。新一届政府要树立强烈的责任意识和紧迫感，把握新机遇，迎接新挑战，推动各项工作再上新台阶。

　　今后五年政府工作的总体要求：高举中国特色社会主义伟大旗帜，坚持以邓小平理论、“三个代表”重要思想和科学发展观为指导，大力实施“科学发展、全面转型、富民强市”战略，着力深化改革开放和强化创新驱动，着力优化经济结构和提高经济增长质量，着力加强节能减排和生态环境保护，着力改善民生和促进社会和谐，不断增强经济发展的内生活力和动力，统筹抓好经济、政治、文化、社会和生态建设，地区生产总值、城乡居民收入比2010年翻一番以上，为提前实现全面建成小康社会的宏伟目标而努力奋斗。

　　新一届政府既承接“十二五”规划，又面对“十三五”发展。我们将深入贯彻落实党的十八大精神，按照市委十一届四次全委会议的要求，努力提升城市综合竞争力，提升城市品质和城乡统筹发展水平，提升全市人民的幸福指数。继续推进产业定位由老工业基地向现代产业基地转变，城市功能由相对单一的工业城市向区域性中心城市转变，动力机制由主要依靠物质资源投入拉动向科技创新驱动转变，发展取向由更多地追求经济增长向更加关注和改善民生转变，增长模式由粗放型增长向集约型增长转变。深入实施产业结构转型升级工程，生态宜居城市建设和城乡统筹发展工程，科教兴市和人才强市工程，文化大市建设工程，以保障和改善民生为重点的社会建设工程，环境综合整治和生态保护工程，不断构筑新优势，谋求新突破，实现新发展。

　　按照“以人为本”理念和“五位一体”总布局的要求，我们将以人民满意作为衡量政府工作的最高标准，认真回应人民群众对美好生活的新期待。加快经济建设，推进发展方式转变和转型升级，把发展成果更多惠及广大群众，建设富裕包头，增强全市人民物质上的富足感。加快政治建设，创造权利公平、机会公平、规则公平的环境，促进政府工作更加高效透明，建设法治包头，增强全市人民权益上的公平正义感。加快文化建设，全面提高公民道德素质，丰富群众精神文化生活，提升地区文化软实力，巩固拓展文明城市建设成果，建设文明包头，增强全市人民精神上的富有感。加快社会建设，加强和创新社会管理，把保障和改善民生放在更加突出的位置，确保人民安居乐业、社会安定有序，建设和谐包头，增强全市人民生活上的幸福感。加快生态文明建设，促进资源节约，完善城市功能，提升城市品位，推进城乡一体化发展，优化生产、生活、生态空间，建设美丽包头，增强全市人民居住上的舒适感。

　　2013年是全面贯彻落实党的十八大精神的开局之年，是实施“十二五”规划承前启后的关键一年，是为提前全面建成小康社会奠定坚实基础的重要一年。我们将按照中央、自治区经济工作会议的部署和要求，全力做好今年各项工作。主要预期目标是：生产总值增长12.5%，地方财政总收入增长13%左右，全社会固定资产投资增长18%以上，城镇居民人均可支配收入增长12.5%，农牧民人均纯收入增长13%，城镇登记失业率控制在3.9%以内。完成自治区下达的节能降耗和主要污染物减排任务。为实现上述目标，我们将重点抓好八个方面工作。

　　一、加快转变经济发展方式，不断提高经济增长质量和效益

　　牢牢把握扩大内需这一战略支点和发展实体经济这一坚实基础，坚持错位发展、延伸发展、集群发展、低碳发展、融合发展，深化改革开放，用高新技术和先进适用技术改造提升传统产业，加快发展战略性新兴产业、现代服务业和农牧业，着力构建传统产业新型化、新兴产业规模化、支柱产业多元化的产业发展新格局。

　　（一）加快工业结构调整。狠抓投资和项目建设。发挥好投资对经济增长的关键作用，在选准方向、优化结构、提高质量和效益的前提下，进一步扩大投资规模。做好项目前期工作，争取国家、自治区更多项目的批复和资金投入。适度增加政府投入，不断扩大市场融资，积极引导民间投资。加大项目建设力度。加快实施包钢综合技改工程，新体系建设项目力争年内投产。加快中国兵器高端装备制造园建设，五万台北奔重卡总装、十万台重卡车架等项目年内投产，铸造、锻造和传动项目尽早开工，新建重卡发动机项目。积极推进包钢氧化矿搬迁和泛海煤化工项目。继续抓好神华煤化工二期和小轿车项目的前期工作，推动早日落地。着力实施国瑞炭谷碳纤维、达茂旗140万千瓦风电、南京高精传动LED蓝宝石衬底、铁路装备和煤机项目，做大高端装备制造、新材料、新能源等新兴产业规模。加快制定稀土产业发展规划，加强行业管理，提高应用水平，促进稀土产业健康发展。加快信息资源整合与开发利用，鼓励云计算技术应用，推进高端软件产品开发。

　　狠抓园区建设。健全完善以奖代补和“共建统管、税收分成”政策，加大园区考核和扶持培育力度。推进稀土高新区与九原工业园区整合，加快达茂旗与白云矿区合作共建工业园区。推进装备制造产业园区晋升为国家级开发区，土右新型工业园区、达茂巴润工业园区晋升为自治区级工业园区。集中力量打造装备制造产业园区成为千亿元级园区，支持稀土高新区更好更快发展，力争使全部工业园区步入营业收入超百亿园区行列，构建优势显著、特色鲜明、带动力较强的产业集群。

　　狠抓企业发展。实施优质企业扶持政策，加快培育一批主业突出、核心竞争力强、行业领先的企业集团，确保包铝、二冶等四户企业营业收入超百亿，使超百亿元企业达到十户。落实扶持中小企业发展的政策措施，积极解决企业融资难、贷款难等问题，为中小企业提供良好服务。支持中小企业转型升级，围绕优势特色产业抓产业延伸，围绕骨干企业做好协作配套，推动大中小企业协调发展。推进工业化和信息化深度融合，支持企业提高智能化和自动化水平。积极争取国家、自治区的政策和资金支持，加大企业减负和治理“三乱”力度，协调解决好企业发展遇到的困难和问题。

　　（二）加快推进服务业提质增效。突出抓好生产性服务业。培育壮大物流企业，加快发展第三方物流。整合物流资源，完善物流园区的信息服务、仓储配送、联运和展示交易功能。加快建设华电物流、华蒙钢铁物流、北方风驰物流港等项目，推动中远洋物流等项目尽快落地。加强金融服务体系建设，积极引进股份制商业银行和其他金融机构，搞好国家开发银行“三融”试点工作，加快包商银行发展，支持村镇银行、小额贷款公司、融资性担保公司发展。有效防范和化解金融风险。培育会计、律师、资产评估、审计、咨询等现代中介业，不断为第三产业发展注入新的活力。

　　大力提升商贸流通业档次。按照新的城市设计要求，加快钢铁大街沿线改造，大力实施金融文化中心、新阳光国际城、茂业天地等商贸项目。深入推进北方国际工业原料城、瑞盛置业国际汽配城、西部五金机电城建设，培育具有较强辐射力的区域营销中心。实施时代广场、维多利新天地等城市综合体建设，着力提升传统服务业层次。

　　有效扩大城乡居民消费。积极创造条件，扩大城乡居民的住房消费，满足群众改善居住条件的刚性需求。深入实施社区商业示范工程和家庭服务体系建设工程，促进便民消费。开拓农村牧区市场，积极推进“万村千乡市场工程”，发展农企居民对接、农批对接、农超对接等流通模式。推进连锁经营、直营配送、电子商务等现代流通方式和新型业态，加快流通体系建设。建设肉菜酒类流通追溯体系和再生资源回收体系，争取进入国家试点城市行列。加强市场监管，规范市场秩序，营造良好消费环境。

　　（三）加快城乡一体化发展。坚持工业反哺农业、城市支持农村牧区和多予少取放活方针，着力推动县域经济发展。加快产业培育、城镇发展和基础设施建设，努力形成以工促农、以城带乡，工农互惠、城乡一体的新型工农、城乡关系。发展特色产业。利用特有条件和优势资源，宜工则工、宜商则商，加快非农产业发展。积极推进都市现代农牧业发展。实施“蔬菜产业提升工程”，稳步发展蔬菜保护地，新增保护地两万亩，达到15.5万亩。强化蔬菜示范园区和种苗基地建设，建立蔬菜加工、物流体系，不断提高质量和效益。实施“马铃薯产业转型工程”，稳定滴灌面积，新增五万亩，达到四十五万亩；新增种薯五万亩，达到二十万亩，打造种薯产业。推进产销加储一体化，提升产业层次。充分发挥我市“群羊”企业的带动作用，实施“肉羊产业提质工程”，继续推进舍饲圈养，加快品种改良，大力发展饲草产业，增强可持续发展能力。实施“奶牛产业增效工程”，稳定饲养数量，加强标准化、规模化奶牛小区（牧场）建设，推进“奶业振兴、苜蓿发展”行动，提升奶业整体发展水平。加快发展农牧业产业化，强化政策、投入、科技、装备、安全五项保障，重点培育规模大、层次高的加工流通企业，规范壮大专业合作组织。充分拓展农牧业功能。积极开发具有包头特色的都市休闲农牧业项目，促进第一和第三产业融合发展。抓好城镇建设。进一步优化旗县区政府所在地城关镇的空间布局，增强城关镇、建制镇的承载能力和辐射带动作用。大力探索小城镇建设发展模式，争取进入国家绿色重点小城镇建设试点行列。推动城乡统筹一体化试点村镇和新农村新牧区示范村镇建设。在集体经济实力较强、改制条件较成熟的城中村、城边村开展新型集体经济股份制改造试点。加快形成工业化和城镇化互动、城镇化和新农村新牧区建设协调发展的格局。加快户籍制度改革。加强基础设施建设。积极争取项目，整合各类资金，推进农村牧区公路、农田水利、电力等基础设施建设，着力在城乡规划、基础设施、公共服务等方面推进一体化。

　　（四）大力推进改革开放。深化草原糖业、明天科技等国企改革，加快厂办大集体改革。完善国有资产管理体制，实现保值、增值。加快财税体制改革，进一步健全公共财政体系。深化科技体制改革，推动科技和经济更紧密结合。探索石拐区资源枯竭型地区转型发展之路。毫不动摇地鼓励、支持、引导非公有制经济发展，努力解决各种影响非公有制经济发展的矛盾和问题。不断放宽市场准入，积极破除阻碍民间投资的体制机制障碍。强化政策支持，全面清理各种歧视性规定，加强服务平台建设，依法保护非公有制企业的合法权益。努力发挥好非公有制经济在稳增长、调结构、惠民生等方面的重要作用，发挥好非公有制经济在繁荣市场、增强经济活力中的重要作用。不断提高开放型经济水平。高度重视招商引资工作，吸引国内外大企业在包头设立地区总部，建立研发机构、生产基地、营销中心和采购中心。加快异地商会、专业商会建设，推进以商招商。加快满都拉口岸建设，大力发展边境贸易和口岸经济，扩大与俄罗斯、蒙古国的合作，鼓励支持有比较优势的企业对外合作或到境外办厂，提高外包工程和劳务合作质量。加强与周边城市的经济协作，积极参与呼包银经济带和呼包鄂城市圈建设，主动承接发达地区产业转移。转变外贸发展方式，优化进出口产品结构，推动我市开放型经济加快发展。

　　二、积极推进宜居城市建设，进一步增强城市的服务功能

　　科学有序地做好城市建设各项工作，继续完善城市功能、优化城市环境、加强城市管理，保持舒展、大气的建设风格，增强城市的吸引力和美誉度，努力把我市建成创业的优先选择、生活的宜居场所。

　　（一）强化规划引导。确定合理的城市规模，从城市空间的尺度、形态、体量和色彩等方面加强立体式规划引导，科学实施经市人大常委会审议通过的钢铁大街———阿尔丁大街等十一项重点区域城市规划设计。完成城市北出口等主要出入口及北梁地区的城市设计。不断完善控规编制成果，加大专项规划编制力度，优化基础设施规划布局。推进新都市区地下“综合管廊”试点规划编制，完成110和210国道户外广告设置规划以及地下管网普查工作。

　　（二）完善基础设施。全年实施城建项目187个，总投资817亿元，年内计划投资268亿元。高质量建设新都市区，加快土右旗中等城市建设，抓好赛汗塔拉城中草原西侧“城中村”迁建、东河北梁地区改造工作。加快完善供热设施布局，建设两座热源厂，满足昆区北部地区、青山区东北部地区各800万平方米热源需求。大力发展热电联产和区域锅炉供热互为补充的供热模式，逐步实现多热源联网运行，解决好热源不足问题。推动循环经济园区建设，促进污泥、餐厨垃圾回收再利用。完善市区垃圾转运站及公厕设施，提高垃圾回收和无害化处理率。加快推进昆河、二道沙河、四道沙河综合整治以及黄河大堤加固，启动东河二期改造治理，不断完善城市防洪和减灾体系。加快园林绿化建设，做好建设路等重要路段夜景亮化工作。

　　（三）加快交通建设。继续推进以国省干线为主的公路建设，确保110国道、包石公路建成通车，开工建设沿黄一级公路，改建希拉穆仁至百灵庙一级公路，配合做好京藏高速包头段南幅建设。继续推动城市立交和地下通道建设，逐步打通哈屯高勒路东西段、青山路东西段、昆河西路等12条道路，努力缓解交通拥堵。建立城市公交政策支持和补贴长效机制，开工建设公交车辆保养基地和两处公交综合站场，开辟、优化公交线路，增加运营里程，购置公交车辆，加强运营管理，不断吸引更多市民选择公交出行，提高公交分担率。推进明安至明登铁路项目建设，加快城市轨道交通项目的规划设计、报批工作。

　　（四）全面加强城市管理。完善城市管理体制，提高精细化管理水平。加强物业管理，提升服务水平。加强综合整治，坚决打击城乡结合部和拆迁改造区域的私搭乱建行为。加强市容环境管理，不断改进城市道路、节点、出入口、旧小区和小街巷市容环境卫生。加强市政设施维修管护，加快城市出入口、重要节点的户外广告整治。加强停车场建设和管理，规范道路停车秩序。加强房地产市场监管，严控市场准入，建立健康规范有序的市场秩序。加强建筑工程管理，确保施工安全和工程质量。

　　三、全面实施创新驱动发展战略，积极培育转型发展的新动力

　　完善科技创新体系，促进创新资源高效配置和综合集成，统筹推进各类人才队伍建设，把全社会智慧和力量凝聚到创新发展上来。全年研究与试验发展经费力争达到55亿元，增长22%，占生产总值的比重达到1.4%。

　　（一）强化企业科技创新主体地位。围绕优势产业、战略性新兴产业和现代农牧业，组织实施重大科技项目二十项。实施企业技术创新引导工程。支持北奔重汽、瑞科稀土、包铝集团创建国家创新型试点企业，新增国家高新技术企业十家，市级以上创新型企业二十家，自治区级和市级企业技术中心十家以上。争取一机集团“中重型传动实验室”建成国家级企业重点实验室。加快推进北师大创意园、清华科技园项目落地。

　　（二）进一步深化产学研合作。积极推动北京大学科技园建设，促进更多的研究成果在我市实现产业化。加快推进中科院、清华大学在我市设立科研分支机构，促进与我市科研院所、高等院校的合作。进一步加强北重特种材料、稀土新材料院士工作站建设，年内力争新增两个院士工作站和两个博士后科研工作站。积极推动建立新的产业技术创新战略联盟，整合聚集科技资源，提升产学研合作层次和水平。

　　（三）建立完善科技创新服务平台。加强科技创新融资平台建设，积极支持科技型中小企业发展。加快建设包头装备制造业科技创新服务平台、世界稀土行业专利信息平台、大型科学仪器设备协作共享平台。促进大学科技园、留学人员创业园、国家级软件园、稀土企业孵化器、科技信息、专利服务等科技中介机构快速发展。

　　（四）深入实施知识产权战略。引导企业把知识产权的创造、管理、应用和保护纳入企业生产经营。推动有实力的企业在关键技术领域形成拥有核心技术的发明专利，专利申请量力争达到2000件。鼓励和引导企业、高校、科研机构积极参与国际标准、国家标准和行业标准的制定、修订工作。开展国家、自治区和包头市三级知识产权制度试点示范和国家企业专利工作站建设，全年新增试点、示范单位和国家企业专利工作站十家左右。

　　（五）全面加强人才队伍建设。广纳英才，加快确立人才优先发展战略，开创人人皆可成才、人人尽展其才的局面。以44个创新团队、257名领军人才为重点，大力实施“5512”专业技术人才创新能力提升工程。完善人才政策，搭建信息平台，营造创业氛围，不断引进各类专业人才，特别是拥有自主知识产权或掌握核心技术的海内外高层次人才。优化人才评价办法，完善事业单位岗位设置管理、公开招聘制度，加大评选表彰力度，形成激励各类人才脱颖而出、积极进取的机制。

　　四、加快文化大市建设，着力增强文化软实力

　　新的发展，要求我们既要有发达的经济，也要有繁荣的文化；既要有经济硬实力，也要有文化软实力。要树立“大文化”发展理念，切实发挥好文化引领风尚、教育人民、服务社会、推动发展的作用，满足人民群众日益增长的精神文化需要。

　　（一）不断加强社会主义核心价值体系建设。积极探索践行社会主义核心价值观的有效形式。深入开展理想信念教育和爱国主义、集体主义、社会主义教育，充分发挥好爱国主义教育基地和图书馆、博物馆等公益性文化设施功能。大力弘扬民族精神和时代精神，在继承“艰苦奋斗、开拓进取、团结拼搏、争创一流”的包头精神前提下，根据新的形势要求，广泛征集各方面意见，提炼形成新的包头精神，激励全市人民争做爱岗敬业、诚实守信、文明有礼、务实进取的模范。加强舆情分析研判，重视网络文化建设管理，坚持正确的舆论导向，形成良好的舆论氛围。

　　（二）全面提高公民思想道德素质。重视加强社会公德、职业道德、家庭美德、个人品德教育，大力弘扬社会主义道德风尚。扎实开展农村民风建设，倡导移风易俗，抵制封建迷信，在全社会形成积极向上的精神追求和健康文明的生活方式。深入开展未成年人思想道德建设，引导未成年人健康成长。深化拓展道德实践活动，继续开展“讲文明树新风”、文明礼仪“六进”活动。深入开展道德领域突出问题专项教育和治理，加强政务诚信、商务诚信、社会诚信和司法公信建设。认真总结全国文明城市创建经验，打造创建活动品牌，推动创建工作常态化。

　　（三）不断丰富人民群众的文化生活。深入挖掘阴山边塞文化、草原文化、敕勒川文化、西口文化、工业文化等资源，打造具有鲜明特色的文化品牌。提升鹿城文化艺术节的举办质量，加强与旅游、商贸相结合，扩大节庆活动影响力。办好春节联欢晚会、新年音乐会、鹿城元宵文化节等文娱活动。提炼各地特色文化，打造国家和自治区级“民间艺术之乡”、“特色艺术之乡”等品牌。继续开展文化艺术惠民“五进活动”，努力把优秀剧目、民族音乐和文化知识送到广大群众身边。积极实施农村牧区电影放映工程，每个行政村放映电影不低于十二场。净化银屏声频，开展“扫黄打非”活动，整治校园周边环境，为青少年健康成长创造良好的社会文化环境。加强公共体育设施建设，广泛开展全民健身运动，促进群众体育和竞技体育全面发展。

　　（四）提高公共文化事业发展水平。完善公共文化服务体系，加快城乡公共文化基础设施建设。打造民族特色文艺精品，编创大型旅游歌舞剧、精品话剧和具有地方特色的电视剧，力争有一批精品创作获得自治区、全国“五个一工程奖”。加大文化遗产保护力度，重视民间工艺、音乐、剪纸、面塑和蒙古族传统文化等非物质文化遗产的挖掘、保护、传承和发扬。加强文化人才队伍建设，鼓励我市大专院校、科研院所、社会培训机构培养文化紧缺人才，选拔专业人才进修学习，建立文化人才库，积极引进高层次、高技能的文化人才，努力培养一批具有影响力的文化骨干。加快文化体制改革，完成国有文艺院团和国有直属影剧院转企改制。推进新闻业务与经营业务分开，建立报业集团和广电传媒公司，实现多元经营。深入推进广播电视户户通工程向农村牧区覆盖，不断提高覆盖率。加快广播电视采编制作播出中心和广播电视发射塔项目建设。

　　（五）提升文化产业竞争力。完善扶持文化产业的政策措施。实施重大文化项目带动战略，推进文化产业招商和上市融资，培育一批骨干企业，发展一批专精特新的中小型文化企业，实施一批文化产业园区建设，支持重点文化企业做大做强。加快建设赛汗塔拉城中草原蒙元文化、南海湖西口文化、九原秦汉文化、秦长城文化、迪士尼消费品迪趣等一批重点文化产业项目，积极推进五当召、美岱召、希拉穆仁景区改造，推进小白河景区项目和包头国家广告产业园区建设，加快申报“中国（包头）少数民族文化产业交易中心”。重点发展演艺娱乐、广播影视、新闻出版以及印刷业，加快发展文化创意、动漫游戏、移动多媒体等新兴文化业态。加强包头国际会展中心的配套设施建设，提升会展业水平，打造“稀土论坛”、“装备制造论坛”等会展品牌。加强文化产业统计工作。引导和鼓励社会力量参与公共文化服务，鼓励支持民间资本进入法律法规许可的文化产业领域。加快重点景区旅游设施建设，开发特色旅游线路和产品，发展旅游新业态，推进文化产业与旅游业深度融合。

　　五、加大社会建设力度，完善基层公共服务和社会管理网络

　　更加注重统筹经济与社会协调发展，不断创新社会管理，推进基本公共服务均等化，使社会服务更加完善，社会管理更加科学，社会参与更加广泛，社会关系更加和谐。

　　（一）努力办好人民满意的教育。合理布局、均衡配置各类教育资源，加快学校标准化和现代化建设，满足适龄儿童就近接受优质义务教育。全面普及高中阶段教育，突出优质学校的示范引领和辐射带动作用，推动高中教育多样化、特色化发展。大力发展公办幼儿园，积极扶持民办幼儿园，全面完成自治区学前教育三年行动计划。积极推动职业教育发展，深入开展校企合作，努力培养各类技能型、实用型人才。进一步加快职教园区建设，形成集教学、科研、实习、服务、生产为一体的多功能职教基地。重点发展民族教育，加快回中新校区建设，年内实现包头蒙中搬迁。支持高等教育内涵式发展，更好地满足经济社会发展对各类专门人才的需求。大力促进教育公平，推动进城务工人员随迁子女平等接受义务教育。支持特殊教育发展。稳定农村牧区教师队伍。加大教师培训力度，提高师德水平和业务能力。全面推进素质教育，培养学生的社会责任感、创新精神和实践能力。

　　（二）提高人民群众健康水平。优化医疗资源配置，加快医疗卫生服务体系建设，更好地满足人民群众多元化医疗需求。建设十个标准化社区卫生服务中心。免费为全市65岁以上老人体检。深化医药卫生体制改革，推进县级公立医院改革试点。完善新农合制度，推进大病商业保险试点。大力实施“名医人才”培养工程，加快农村牧区医疗卫生队伍建设。深入开展爱国卫生运动，巩固提高国家卫生城市创建成果。坚持计划生育基本国策，提高出生人口素质，促进人口长期均衡发展。

　　（三）加强和创新社会管理。完善矛盾纠纷排查和预警机制，抓好矛盾调处和源头治理。落实信访工作责任制，不断化解各类信访积案。进一步建立健全社会稳定风险评估机制。强化流动人口服务管理，抓好闲散青少年、农村牧区留守儿童等特殊人群服务管理。加强“大调解”机制建设，推进人民调解、行政调解和司法调解有机结合。建立健全民意诉求表达机制，拓宽群众意愿表达渠道。健全安全生产责任制，突出抓好重点行业和领域安全生产，杜绝重特大事故发生。深入推进平安建设，积极防范和依法打击违法犯罪活动，切实保障人民群众生命财产安全。加强食品药品监管，加大违法违规案件查处力度，让人民群众吃得放心、用得安心。加强社区建设，促进基层管理力量整合联动。全面推广“精街道强社区”试点经验，不断提高社区管理水平。积极发挥工青妇等群团组织的作用。大力弘扬志愿精神，完善志愿者服务制度。全面贯彻党的民族宗教政策，广泛开展民族团结进步宣传和创建活动，促进各民族和睦相处、和衷共济、和谐发展。发挥宗教界人士和信教群众在经济社会中的积极作用。深入开展国防教育，加强国防动员和后备力量建设。扎实开展双拥共建，巩固和发展军政军民团结，促进军民融合式发展取得新成效。深化爱民固边战略，提升边防管理服务水平。加强应急管理与处置能力建设，最大限度地减少突发事件造成的危害。

　　六、着力保障和改善民生，进一步提高人民群众的生活水平

　　从解决人民群众最关心、最直接、最现实的利益问题入手，切实完善保障和改善民生的各项政策，多谋民生之利，多解民生之忧，努力让人民过上更好的生活。继续为人民群众兴办16件好事实事。

　　（一）切实加强就业工作。加大小额担保贷款扶持力度，加强创业服务体系建设，充分发挥创业带动就业的倍增效应。切实抓好高校毕业生、就业困难人员等重点群体就业。继续推进职业技能培训，提升创业能力，增强就业稳定性。强化就业服务实名制管理，加强对重点行业、重点企业失业动态监测。积极推进劳动关系三方协调机制建设，加强劳动保障监察“两网化”管理和劳动争议仲裁实体化建设，切实维护劳动者合法权益。年内实现城镇新增就业4.1万人。

　　（二）不断增加城乡居民收入。按照国家和自治区有关政策，推行企业工资集体协商制度，完善企业工资正常调整机制。认真执行最低工资标准。做好企业退休人员基本养老金调整工作。着力抓好农牧民增收工作。全面落实国家和自治区各项惠农惠牧政策，完善我市“三农”扶持政策，促进农牧民政策增收。充分挖掘农牧业内部增收潜力，推动农牧业转型升级，增加经济效益，促进农牧民产业增收。加强中介组织、经纪人和农牧民培训，提高农牧民素质，强化劳务输出，增加农牧民工资性收入。加大扶贫工作力度，投入扶贫资金1.2亿元以上，全面实施整村推进、产业化扶贫、集中连片、科技扶贫等项目，解决2.5万贫困人口的生产生活问题。继续组织社会力量集中帮扶固阳县。

　　（三）进一步做好社会保障工作。做好各项社会保险扩面、核定和征缴清欠工作，不断提升社会保险保障能力。适时调整各项社会保险待遇水平，确保按时足额支付。城镇低保标准提高15元，达到每人每月520元，农村牧区低保标准提高144元，达到每人每年4624元。全市集中和分散供养“五保户”供养标准提高800元和516元，达到每人每年7500元和4816元。积极探索医疗保险付费方式改革，推动包钢、一机、包铝封闭运行企业纳入医疗保险市级统筹。加大老工伤人员纳入工伤保险统筹的资金征缴力度，将机关和参公事业单位职工纳入工伤保险统筹管理。大力发展社区养老服务，新建三十个社区日间照料中心。大力提高蔬菜等农副产品自给能力，加强购销衔接与供应保障，保障农副产品市场供应和价格基本稳定。完善社会救助标准与物价上涨挂钩的联动机制，及时向困难群众发放临时价格补贴。农村牧区低保家庭冬季取暖补贴达到每户每年400元。促进残疾人事业发展，积极发挥红十字会的作用。继续加大保障性住房建设力度，切实提高管理水平，基本建成保障性住房2万套。

　　七、加强生态建设和环境保护，努力创建国家环保模范城市

　　推进生态建设，加强环境保护，继续组织实施好“蓝天、绿水、青山、湿地、大草原”工程，实现人口资源环境相均衡、经济社会生态效益相统一，着力构建资源节约型、环境友好型社会。

　　（一）节约集约利用资源。不断降低能源、水、土地消耗强度。落实国家万家企业节能低碳行动，强化节能执法，加快推进企业能源审计和节能规划编制，完成国家要求的进度目标。合理利用环境容量，加快初级加工产业向城区外转移，淘汰低端产业和落后产能，实施城区“退二进三”、“退低进高”，基本完成“三片两线”综合整治的预定任务。加强集中式饮用水源地水质安全监管，尽快推进保护区范围内排污口及违章建筑清理整顿。实施再生水利用工程，提高中水回用水平。严守耕地保护红线，推进工业项目向园区集中。合理开发矿产资源，加强尾矿库保护，加快铌、钍等战略资源开发利用。

　　（二）继续加强环保工作。实施“污水零排放”计划，持续治理工业废水。全力推进九原、西郊等四个污水处理厂新建、扩建，加快污水收集管网建设。强力推进“蓝天工程”，实施PM2.5监测。加强重点污染源的治理，做好华业特钢周边企业、西河楞、万水泉地区的企业与煤场的搬迁。加快扬尘污染综合整治，解决料场堆场、露天矿山开采加工、建筑工地等造成的扬尘污染问题，完成东河区公积板、土右旗大城西煤场等区域环境整治工作。推行机动车黄绿标管理制度，逐步淘汰2005年以前注册运营的“黄标车”。加快实施“固体废物资源化利用和污染控制”工程，完善固废综合利用政策措施，提高工业固废处置利用水平。加大环境执法监察力度，坚决清理整治违法排污企业。严格日常巡查和重点督办，保障污染治理设施稳定运行，确保环境安全。继续大力度、大投入推进国家环保模范城市创建工作，加快完成创模重点工程，不断改善环境质量。

　　（三）大力推进生态建设。加强生态保护宣传教育，提升生态文明建设水平。完成国家重点林业造林任务38.5万亩和大青山南坡绿化二期工程1万亩。推进大青山生态供水配套工程建设，大力发展滴灌造林，打造设施林业。建设村屯绿化示范区，继续实施五十六个村屯的绿化工作。实施绿色通道工程，逐步绿化市域内高速公路、国道、省道、县道，促进城区、城郊结合部和村屯绿化“无缝对接”。强化义务植树责任，引入社会资本参与林业建设，加强后期管护，提高造林成活率。进一步加快大青山国家级自然保护区建设和管理，打击各类违法行为，完成核心区内生态移民和生态恢复二期工程。全面开展黄河国家湿地公园建设，加快实施包头黄河湿地生态系统定位观测研究站项目，提高湿地保护水平，发挥好湿地碳汇作用。进一步巩固围封禁牧成果，深入落实草原生态补奖机制，强化禁牧管护，妥善解决转移牧民后续的生产生活问题，实现草原增绿和农牧民增收。

　　八、不断提高依法行政水平，切实优化政务环境

　　加快转变政府职能，改进工作作风，提高行政效能和服务水平，增强政府执行力和公信力，更好地服务于经济社会发展，建设高效透明责任政府。

　　（一）严格依法行政。全面执行人民代表大会及其常委会的决议，依法接受市人大的法律监督和工作监督，自觉接受市政协的民主监督，坚持定期向市人大及其常委会报告工作和向市政协通报情况。认真听取民主党派、工商联、无党派人士和各人民团体的意见。建立健全协商民主机制。认真做好人大代表议案、建议和政协提案的办理工作。严格依照法定权限和程序行使权力、履行职责。健全重大事项专家咨询、公众参与、集体决策制度。继续清理、精简审批事项，严格规范行政审批行为。建立完善并联审批工作机制，推行“一站式”审批。深化政务公开，完善各类公开办事制度，推广电子政务，提高政府工作透明度。

　　（二）加强效能建设。各级行政机关及其工作人员要增强服务意识，确保政令畅通。对中央、自治区及市委的决策部署，做到有令必行、有禁必止，绝不允许上有政策、下有对策。建立完善各项工作制度，全面落实岗位责任制和责任追究制，致力解决工作不实、责任不强、落实不力等问题。强化督查考核机制，更好地把目标与责任、督促与落实、考核与奖惩结合起来，不断提高抓落实的成效。贯彻落实中央出台的八项规定，转变工作作风，改进文风会风。健全公务员管理制度，完善考核评价机制，强化职业道德建设，提升公务员队伍整体素质。

　　（三）坚持廉洁从政。全面推进惩治和预防腐败体系建设，做到干部清正、政府清廉、政治清明。规范公共资源交易，加快公共资源交易平台建设。重视新闻舆论和社会公众监督，支持监察、审计部门依法独立监督。深入开展经济责任审计，强化对权力运行的制约和监督。加大对财政性投资的监管力度，严格控制公务消费。厉行勤俭节约，严格执行住房、车辆配备等有关工作和生活待遇的规定。开展好以为民务实清廉为主要内容的群众路线教育实践活动，着力解决人民群众反映的突出问题。树立正确的政绩观，增强公仆意识，真正做到为人民服务，对人民负责，受人民监督，让人民满意。

　　各位代表！实现包头未来五年发展的美好蓝图，需要我们脚踏实地，从现在做起。让我们紧密团结在以习近平同志为总书记的党中央周围，在自治区党委、政府和包头市委的领导下，凝聚全市人民的智慧和力量，振奋精神，开拓进取，扎实工作，在实现科学发展新跨越的伟大实践中再创辉煌！
